# A MiRNA Signature for Defining Aggressive Phenotype and Prognosis in Gliomas

**DOI:** 10.1371/journal.pone.0108950

**Published:** 2014-10-03

**Authors:** Raffaela Barbano, Orazio Palumbo, Barbara Pasculli, Marco Galasso, Stefano Volinia, Vincenzo D'Angelo, Nadia Icolaro, Michelina Coco, Lucia Dimitri, Paolo Graziano, Massimiliano Copetti, Vanna Maria Valori, Evaristo Maiello, Massimo Carella, Vito Michele Fazio, Paola Parrella

**Affiliations:** 1 Laboratory of Oncology, “IRCCS Casa Sollievo della Sofferenza”, San Giovanni Rotondo, FG, Italy; 2 Laboratory of Genetics, “IRCCS Casa Sollievo della Sofferenza”, San Giovanni Rotondo, FG, Italy; 3 Department of Biosciences, Biotechnology and Biopharmaceutics, University of Bari “Aldo Moro”, Bari, Italy; 4 LTTA, Department of Morphology, Surgery and Experimental Medicine, Human Anatomy Branch, University of Ferrara, Ferrara, Italy; 5 Department of Neurosurgery, “IRCCS Casa Sollievo della Sofferenza”, San Giovanni Rotondo, FG, Italy; 6 Laboratory of Oncology, “IRCCS Casa Sollievo della Sofferenza”, San Giovanni Rotondo, FG, Italy; 7 Department of Pathology, “IRCCS Casa Sollievo della Sofferenza”, San Giovanni Rotondo, FG, Italy; 8 Unit of Biostatistics, “IRCCS Casa Sollievo della Sofferenza”, San Giovanni Rotondo, FG, Italy; 9 Department of Oncology, “IRCCS Casa Sollievo della Sofferenza”, San Giovanni Rotondo, FG, Italy; 10 Laboratory of Genetics, “IRCCS Casa Sollievo della Sofferenza”, San Giovanni Rotondo, FG, Italy; 11 CIR Laboratory for Molecular Medicine and Biotechnology, University Campus Biomedico, Rome, Italy; Johns Hopkins University, United States of America

## Abstract

Gliomas represent a disparate group of tumours for which there are to date no cure. Thus, there is a recognized need for new diagnostic and therapeutic approaches based on increased understanding of their molecular nature. We performed the comparison of the microRNA (miRNA) profile of 8 WHO grade II gliomas and 24 higher grade tumours (2 WHO grade III and 22 glioblastomas) by using the Affymetrix GeneChip miRNA Array v. 1.0. A relative quantification method (RT-qPCR) with standard curve was used to confirm the 22 miRNA signature resulted by array analysis. The prognostic performances of the confirmed miRNAs were estimated on the Tumor Cancer Genome Atlas (TCGA) datasets. We identified 22 miRNAs distinguishing grade II gliomas from higher grade tumours. RT-qPCR confirmed the differential expression in the two patients' groups for 13 out of the 22 miRNAs. The analysis of the Glioblastoma Multiforme (GBM) and Lower Grade Glioma (LGG) datasets from TCGA demonstrated the association with prognosis for 6 of those miRNAs. Moreover, in the GBM dataset miR-21 and miR-210 were predictors of worse prognosis in both univariable and multivariable Cox regression analyses (HR 1.19, *p* = 0.04, and HR 1.18, *p* = 0.029 respectively). Our results support a direct contribution of miRNAs to glioma cancerogenesis and suggest that miR-21 and miR-210 may play a role in the aggressive clinical behaviour of glioblastomas.

## Background

Gliomas account for approximately 70% of all malignant primary brain tumours diagnosed in adults. They are currently classified by the World Health Organization (WHO) according to their morphological resemblance to the respective glial cell types, cytoarchitecture and immunohistological marker profile [1]. The vast majority of gliomas are astrocytomas or oligodendrogliomas or a mixture of these two types (oligoastrocytomas). In addition, the WHO classification distinguishes four grades (I, II, III and IV) for astrocytomas and two grades (II and III) for oligodendrogliomas and oligoastrocytomas. The grade IV astrocytoma, also known as glioblastoma multiforme (GBM), is the most common and aggressive form, with a median survival of only 12 to 15 months as compared to 2 to 5 years for patients with anaplastic gliomas (WHO III) and 6–8 years for low grade tumours (WHO I and II) [1].

Currently, the key issues challenging research on glioma include determination of molecular events initiating glioma (gliomagenesis) and involved in the progression from slow growing, well differentiated neoplasms to rapidly growing, highly anaplastic forms. The answers to these questions are crucial to characterize the disease in its neurobiological context and to develop novel and more effective therapies [1].

MicroRNAs (miRNAs) are small non-coding RNAs, of 18 to 25 nucleotides in length, involved in the post transcriptional regulation of gene expression. miRNAs interact with their coding mRNA targets to prevent translation into proteins either by degradation or, more frequently, by direct and imperfect binding to the 3′-untranslated region (3′UTR) and mRNA exonucleolytic decay. Because of the functional role of miRNAs in a wide array of biological processes, including cell proliferation, differentiation and apoptosis, deregulation of miRNA expression represents a hallmark of cancer, where they can act either as oncogenes or tumour suppressors contributing to initiation and progression of cancer [2].

In the attempt to identify molecular determinants associated with aggressive behaviour in gliomas, we compared the miRNA expression profile of grade II gliomas, characterized by a more favourable clinical behaviour, and grade III+IV tumours characterized by the worst clinical outcome. We identified 13 miRNAs differentially expressed in grade II as compared with grade III+IV gliomas. The analysis of the Glioblastoma Multiforme (GBM) and Lower Grade Glioma (LGG) datasets from The Cancer Genome Atlas (TCGA) demonstrated the association with prognosis for 6 of those miRNAs. In particular, in the GBM dataset miR-21 and miR-210 were predictors of worse prognosis in both univariable and multivariable Cox regression analyses.

## Materials and Methods

### Samples Selection

We evaluated 32 glioma samples treated by surgery at the Department of Neurosurgery of the IRCCS “Casa Sollievo della Sofferenza” San Giovanni Rotondo, FG (Italy). All tissue samples were snap-frozen in liquid nitrogen after resection and stored at −80°C until use. All cases were pathologically classified according to the WHO system. Both clinical and follow-up data were provided by the Department of Oncology of the same institute (Table 1). The study was approved by the Ethic Committees of the IRCCS “Casa Sollievo della Sofferenza”. All human materials used in the study were collected according to the guidelines of the local ethical committee. Prior written and informed consent was obtained from each of the patients in accordance with Institutional Guidelines. As control, commercially available RNAs (Agilent Technologies, Waldbronn, Germany) from 4 healthy individuals including different brain regions (parietal cortex, frontal cortex, occipital cortex and striatum) were used.

**Table 1:** Clinico-pathological characteristics of patients cohort.

Characteristics		III+IV Grade (n = 24)		II Grade (n = 8)	
Age	Median		61 years		32 years
	(IQR)		(54–72)		(29–42)
Gender	Male	15	(62%)	8	(100%)
	Female	9	(38%)	0	(0%)
Grade	II	0	(0%)	8	(100%)
	III	2	(8%)	0	(0%)
	IV	22	(92%)	0	(0%)
MGMT	UM	11	(46%)	3	(38%)
	M	13	(54%)	5	(62%)
IDH1	wt	23	(96%)	1	(12%)
	mut	1	(4%)	7	(88%)
IDH2	wt	24	(100%)	8	(100%)
	mut	0	(0%	0	(0%)
Progression	Yes	23	(96%)	3	(25%)
	No	1	(4%)	5	(75%)
Status	Alive	1	(4%)	6	(75%)
	Dead	23	(96%)	2	(25%)
Time to progression	Median		10 months		48 months
	(IQR)		(5–14)		(33–54)
Overall Survival	Median		16 months		60 months
	(IQR)		(12–24)		(39–60)

### DNA and RNA extraction

Nucleic acids were extracted from frozen sections after ensuring by light microscopic evaluation on a hematoxylin and eosin stained section that tumour sample contained more than 70% neoplastic cells. DNA was extracted from one half of the frozen specimen as previously described [3]. The remaining part was carefully and mechanically homogenized in TRIzol reagent according to the manufacturer's instructions (Life Technologies, Foster City, CA). Integrity and purity of small RNAs and total RNA were measured by using Agilent 2100 Bioanalyzer (Agilent Technologies, Waldbronn, Germany) and only RNAs with RIN (RNA Integrity Number) ≥7.0 were subsequently processed. Nucleic acids concentration was quantified by the absorbance measurement at 260 and 280 nm using the NanoDrop 1000 Spectrophotometer (NanoDrop Technologies, Berlin, Germany).

### Microarray analysis

500 ng of RNA were labelled by using the 3DNA Array Detection FlashTag RNA Labeling Kit according with manufacturer's instruction and analyzed by the GeneChip miRNA v. 1.0 Array (Affymetrix, Santa Clara, CA) which contains 46,228 probes comprising 7,815 probe sets and covers 71 organisms including 848 human miRNAs derived from the Sanger miRBase and miRNA database v11 (April 15, 2008, http://microrna.sanger.ac.uk). Briefly, poly (A) tailing was first carried out at 37°C for 15 min. in a volume of 15 ml reaction mix, which contained 1× Reaction Buffer, 1.5 ml MgCl_2_ [25 mM], 1 ml ATP Mix diluted 1∶500 and 1 ml PAP enzyme. Subsequently, Flash Tag Ligation was performed at room temperature for 30 min by adding 4 ml of 5× Flash Tag Ligation Mix Biotin and 2 ml T4 DNA Ligase into the 15 ml of reaction mix. To stop the reaction, 2.5 ml of Stop Solution was added. Finally, samples were hybridized on the arrays which were washed and stained with the Affymetrix Fluidics Station 450 and scanned with the Affymetrix GeneChip Scanner 3000 7G using the Command Console software (Affymetrix, Santa Clara, CA). Data analysis was performed by using Partek Genomic Suite 6.4 software as follows: raw intensity data (.CEL files) were imported by setting up robust multi-array analysis (RMA) background correction, quantile normalization, and log transformation; Principal Component Analysis (PCA) were performed as it is an excellent method for visualizing high-dimensional data and underlie outliers samples; analysis of variance (ANOVA) was performed in order to generate a comprehensive list of differential expressed miRNAs setting a significative *p*-value≤0.01 and a fold change cut-off of 2. The array data have been deposited at the ArrayExpress archive, accession number E-MTAB-1840.

### Quantitative Reverse Transcription Polymerase Chain Reaction (RT-qPCR) analysis

Single-stranded cDNA was synthesized from 5.5 ng of total RNA using 50 nM specific stem-loop RT primers for selected miRNAs (Life Technologies, Foster City, CA) and endogenous control RNU48 (P/N 4373383, Life Technologies, Foster City, CA), according to manufacturer's instructions (Table S1). RT positive and negative controls were included in each batch of reactions. A relative quantification method with standard curve was developed to determine miRNA expression in tissues [4]. PCR fragments for each of the 22 miRNAs and RNU48 endogenous control were generated using TaqMan miRNA assay, cloned in the StrataClone PCR Cloning Vector pSC-A (Stratagene, Santa Clara, CA) and introduced in StrataClone SoloPack Competent Cells. After isolation, plasmid DNA concentration was quantified by spectrophotometric measurement and plasmid copy number was calculated using the following formula: ((X µg/µl plasmid DNA)/(plasmid and insert length)×660 g/mole)×6.023×10^23^ = Y molecular number/µl. X represents the concentration of recombinant plasmid DNA, 660 g/molecule the average MW of a double-stranded DNA molecule, and Y represents copy number. Five plasmid dilutions for miRNA-containing pSC-As and RNU48-containing pSC-A (range from 1×10^6^ copies to 1×10^2^ copies) were used to construct each of the five points calibration curves. Real-time PCR reactions were set up in triplicate in 384-well plates using Taqman 2× Universal PCR Master Mix, No AmpErase UNG (Life Technologies, Foster City, CA) and run in an ABI Prism 7900 HT according to the manufacturer's instructions. Calibration curves were constructed by plotting the threshold cycle (Ct) versus logarithm of the copy number and the analysis was performed by using SDS 2.4 (Life Technologies, Foster City, CA).

### Fluorescence-based direct sequencing analysis

Primer pairs were designed to amplify a fragment spanning the catalytic domain of IDH1 including codon 132, *IDH2* codon 172, and exons 5–8 of *TP5*3 gene (Table 2). Sequence reactions were performed on an automated sequencer (ABI 3100; Life Technologies, Foster City, CA) using the ABI-PRISM Big-Dye Terminator Cycle Sequencing Ready Reaction kit (Life Technologies, Foster City, CA).

**Table 2:** miRNAs differentially expressed among TCGA subclasses sorted by the *p*-value of the univariable test.

Symbol	p-value	FDR	Class 1*	Class 2*	Class 3*	Class 4*	Class 5*	Class 6*	Pairwise significant
hsa-miR-155	<1e-07	<1e-07	554.65	317.69	423.14	92.57	265.23	365.16	(2, 1), (3, 1), (4, 1), (5, 1), (6, 1), (2, 3), (4, 2), (5, 2), (2, 6), (4, 3), (5, 3), (6, 3), (4, 5), (4, 6), (5, 6)
hsa-miR-21	<1e-07	<1e-07	27442.3	15287.98	17883.53	669.96	12182.95	15221.63	(2, 1), (3, 1), (4, 1), (5, 1), (6, 1), (2, 3), (4, 2), (5, 2), (6, 2), (4, 3), (5, 3), (6, 3), (4,5), (4, 6), (5,6)
hsa-miR-210	<1e-07	<1e-07	988.22	399.05	697.26	124.73	457.88	513.53	(2, 1), (3, 1), (4,1), (5, 1), (6,1), (2, 3), (4,2), (2, 5), (2,6), (4, 3), (5,3), (6, 3), (4,5), (4, 6), (5,6)
hsa-miR-219-2-3p	<1e-07	<1e-07	656.5	2921.88	841.23	8532.12	1460.9	517.65	(1, 2), (1, 3), (1, 4), (1, 5), (6, 1), (3, 2), (2, 4), (5, 2), (6, 2), (3, 4), (3, 5), (6, 3), (5, 4), (6, 4), (6, 5)
hsa-miR-22	<1e-07	<1e-07	4109.27	2876.27	3375.01	2715.57	1820.22	2529.99	(2, 1), (3, 1), (4, 1), (5, 1), (6, 1), (2, 3), (4, 2), (5, 2), (6, 2), (4, 3), (5, 3), (6, 3), (5, 4), (6, 4), (5, 6)
hsa-miR-223	<1e-07	<1e-07	1747	994.03	943.95	415.67	579.75	845	(2, 1), (3, 1), (4, 1), (5, 1), (6, 1), (3, 2), (4, 2), (5, 2), (6, 2), (4, 3), (5, 3), (6, 3), (4, 5), (4, 6), (5, 6)
hsa-miR-16	<1e-07	<1e-07	2997.52	2517.15	2439.92	1239.42	2915.65	2906.95	(2, 1), (3, 1), (4, 1), (5, 1), (6, 1), (3, 2), (4, 2), (2, 5), (2, 6), (4, 3), (3, 5), (3, 6), (4, 5), (4, 6), (6, 5)
hsa-miR-105	3.67e-05	3.98e-05	54.26	55.33	54.57	57.48	56.05	54.8	(1, 2), (1, 3), (1, 4), (1, 5), (1, 6), (3, 2), (2, 4), (2, 5), (6, 2), (3, 4), (3, 5), (3, 6), (5, 4), (6, 4), (6, 5)
hsa-miR-451	0.5042	0.504	1007.05	760.26	892.38	623.59	789.01	890.63	(2, 1), (3, 1), (4, 1), (5, 1), (6, 1), (2, 3), (4, 2), (2, 5), (2, 6), (4, 3), (5, 3), (6, 3), (4, 5), (4, 6), (5, 6)

*Class 1: Astrocytic; Class 2: Neural; Class 3 Neuro-mesenchimal; Class 4: Normal; Class 5: Oligoneural; Class 6: Radial glial.

For each class the geometric mean is shown. The ‘Pairwise significant’ column shows pairs of classes with significantly different gene expression at alpha = 1. Class labels in a pair are ordered (ascending) by their averaged gene expression.

### TCGA miRNA dataset and patients' information

miRNA expression data and corresponding clinical information for the Glioblastoma Multiforme (GBM) and Lower Grade Gliomas (LGG) datasets were downloaded from The Cancer Genome Atlas (TCGA) data portal in January and June 2013 respectively. The level 3 data for the 481 glioblastoma patients and 221 LGG patients were quartile normalized and log2 transformed. From the GBM dataset, we selected the 191 patients with overall survival (OS) information and miRNA-based subtype classification [5]. Patients showing OS less than 30 days were excluded from the analysis, since they may have died for disease unrelated reason. Complete data for multivariable analysis were available for 135 patients. For the LGG dataset, information regarding tumour grade were available for 115 tumours. Since grade II (n = 57) and grade III (n = 58) gliomas differ substantially in prognosis, survival analyses were performed on each subgroup separately. Clinical covariates for the TCGA GBM and LGG datasets are summarized in Tables S3a–c.

### Statistical Analyses

Patients' baseline characteristics were reported as median and interquartile range (IQR) or frequencies and percentages for continuous and categorical variables, respectively. For RT-qPCR analysis comparisons among NBT, grade II and grade III+IV groups were made using the Kruskall-Wallis test and Mann-Whitney test. The association between continuous miRNA expression and OS was carried out using univariable Cox regression analysis. Risks were reported as hazards ratios (HR) along with their 95%CI. Furthermore, overall survival curves were estimated using Kaplan-Meier method and were graphically reported [6]. The analyses were performed with BRB-Array Tools - R/BioConductor (version 2.10).

For the multivariable analysis, the Cox proportional hazard model was applied, and a backward stepwise selection procedure (Wald) was used to identify miRNAs with independent prognostic value. All reported *p*-values were two-sided. The analyses were performed using SPSS (version 12.1).

## Results

### Patients Characteristics

We analyzed 8 WHO grade II gliomas, 2 anaplastic astrocytomas (WHO grade III), 22 glioblastomas (WHO grade IV), and 4 Normal Brain Tissues (NBT). Among the grade II gliomas, 1 was an astrocytomas, 4 oligoastrocitomas and 3 oligodendrogliomas (Table S4). All patients included in the study were subjected to resection of a newly diagnosed glioma. Three of the glioblastoma patients underwent to a second tumour resection at recurrence. Post-surgery treatments were performed according to Stupp protocol [7]. Patients' clinicopathological data are summarized in Table 1.

### Molecular characterization of gliomas

Tumour samples were analyzed for *IDH1*, *IDH2* and *TP53* mutational status. *IDH1*-R132H/C mutations were found in 7 out of 8 WHO II grade tumours (88%, R132H in six samples and R132C in one sample), 1 out of the 2 anaplastic astrocytomas (50%, R132C), and as expected by the low frequencies reported in literature in none of the GBMs (0%) [8], [9]. No mutations were found in *IDH2* codon 172. Four nucleotide changes were demonstrated in the *TP53* gene in the grade III+IV group and all of them were polymorphic variants. *MGMT* status was previously determined by using Quantitative Methylation Specific PCR (QMSP): aberrant promoter methylation was found in the 68.75% of the 32 glioma patients [10].

### Microarray profile of gliomas

**Figure 1:**
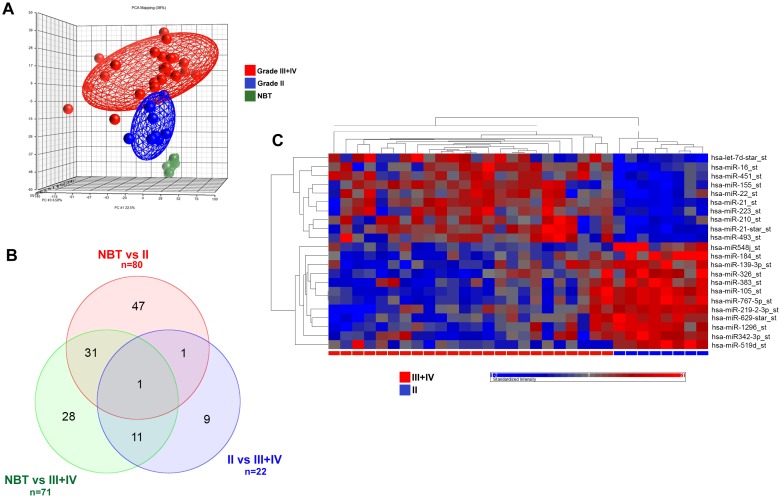
miRNA expression analysis of gliomas. A) Global views of gene expression by using the Principal Component Analysis (PCA) between grade III+IV tumours (HGG, red), and grade II gliomas (LGG, blue) samples and normal brain tissues (NBT, green). The analysis was performed by Partek Genomic Suite software using default setting that includes a threshold to remove low background level intensities. PCA percent mapping on the top of the plot indicates the explained variability on the first coordinates. B) Venn diagrams summarizing the number of miRNAs found to be differentially expressed by comparing grade II gliomas and NBT (green), grade II and grade III+IV gliomas (blue), grade III+IV gliomas and NBT (red). Also, number for conjoint (and non-conjoint) differentially expressed miRNAs are indicated. C) Hierarchical clustering analysis of miRNA expression profile for 8 grade II (blue) and 24 grade III+IV gliomas (red). Columns represent tissue specimens and rows miRNAs.

The 32 glioma samples and 4 normal brain tissues (NBT) were profiled on the Affymetrix GeneChip miRNA Array v. 1.0. PCA analysis demonstrated that miRNA profiling clearly differentiates pathological samples from NBT (Figure 1A). By using ANOVA model, 80 miRNAs were found differentially expressed in grade II gliomas vs NBT, 71 in grade III+IV gliomas vs NBT, whereas only 22 miRNAs clearly differentiated grade III+IV gliomas from grade II tumours (Table S5). In addition, logical relations resulting from our three-sets Venn Diagram show a subgroup of 9 not-shared miRNAs which coherently differentiates grade II and grade III+IV (Figure 1B). Hierarchical clustering of the 22 miRNAs identified by comparing grade II and grade III+IV is shown in Figure 1C.

### miRNA analytical validation by RT-qPCR

**Figure 2:**
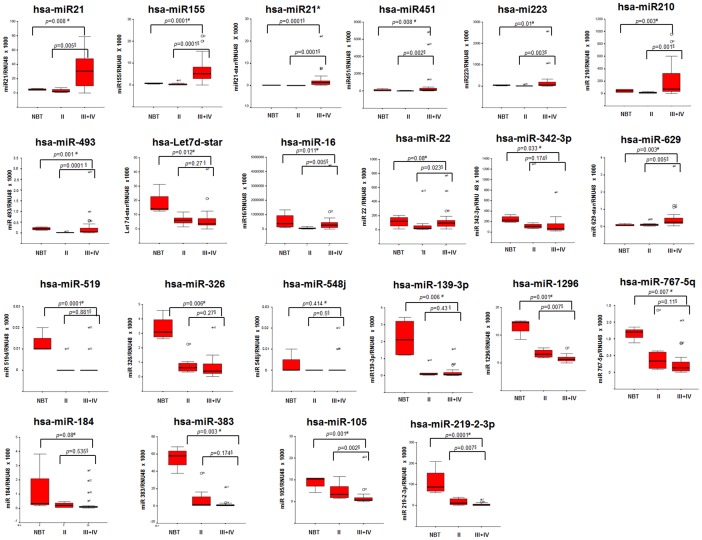
Validation of microarray data by qRT PCR analysis. The 32 glioma samples and 4 NBT were analyzed by RT-qPCR for the expression of the 22 miRNAs identified by microarray profiling. Boxplots of miRNAs expression in normal brain tissues (NBT), WHO grade II gliomas (II) and WHO grade III and IV gliomas (III+IV). Expression levels are expressed as: target miRNA/RNU48 multiplied by 1000. The boxes mark the interquartile range, (interval between the 25th and 75th percentile). The lines inside the boxes denote median values, the symbol * denote the outliers. #NBT vs II vs III+IV comparison by Kruskall Wallis Test; §II vs III+IV comparison by Mann Whitney test.

To confirm the differential expression of the 22 miRNAs identified by array profiling, the 32 samples and 4 NBT were analyzed by RT-qPCR. Standard curves were built by plotting the threshold cycle (Ct) values against the log^10^ of the copy number and fitting by linear least square regression. The level of miRNA expression in each sample was determined as the ratio of the miRNA copy number to the RNU48 copy number and then multiplied by 1000 for easier tabulation (miRNA/RNU48×1000). A statistically significant differential expression among NBT, grade II and grade III+IV subgroups (Kruskall Wallis Test) was demonstrated for 19 out of the 22 miRNAs (Figure 2). In addition, 13 out of the 22 miRNAs (miR-1296, miR-21, miR-155, miR-21-star, miR-451, miR-223, miR-210, miR-493, miR-16, miR-22, miR-629-star, miR-105, and miR-219-2-3p) showed a significant different expression in grade II tumours as compared with grade III+IV (Mann Whitney Test). The remaining miRNAs (miR-519d, miR-326, miR-139-3p, miR-767-5p, let7d-star, miR-342-3p, and miR-383) were differentially expressed only in grade II gliomas as compared with NBT (Mann Whitney Test).

### Prognostic performance of confirmed miRNA on the TCGA dataset

To determine whether the 13 miRNAs identified by our analysis as differentially expressed in grade II tumours as compared with grade III+IV gliomas are associated with prognosis, we evaluated their prognostic performance on two independent patient cohorts from the TCGA datasets. In the GBM dataset the analysis was limited to 9 miRNAs, since miR-1296, miR-21-star, miR-629-star and miR-493 were not included in the Agilent platform used in the study (Table 2). All the 13 miRNAs were instead assessed in the recently published LGG dataset since included in the Illumina analytical platform.

**Figure 3:**
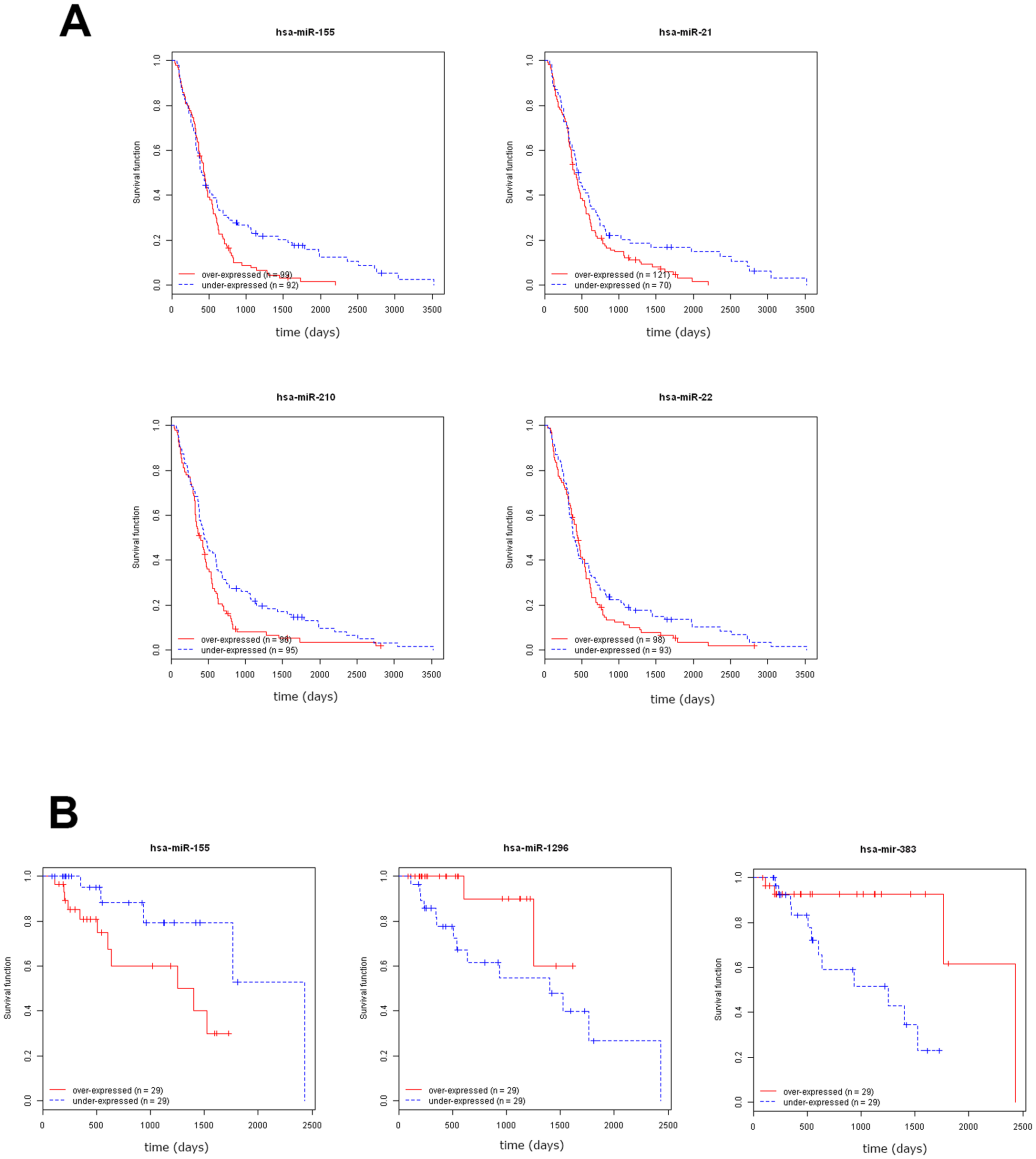
Relationship between miRNAs expression and survival in glioma patients. A) Kaplan-Meier curves showing the relationship of miR-155, miR-21, miR-210 and miR-22 with Overall Survival in patients from the GBM dataset. B) Kaplan- Meier curves showing the relationship of miR-155, miR-1296, and miR-383 with Overall Survival in Grade III glioma patients from the LGG dataset.

In tumours selected from the GBM dataset (n = 191), all 9 miRNAs showed a significant differential expression in the GBM prognostic subclasses defined by Kim *et al* (astrocytic, neural, neuromesenchimal, oligoneural and radial glial). In particular, miR-155, miR-21, miR-210, miR-223 and miR-22 showed the highest expression and miR-219-2-3p the lowest expression in the astrocytic subgroup characterized by the worst prognosis. In univariable Cox regression analysis, overexpression of miR-21 (HR 1.26; 95%CI 1.06–1.48, *p* = 0.007), miR-210 (HR 1.16; 95%CI 1.01–1.33, *p* = 0.038) miR-22 (HR 1.24; 95%CI 1.03–1.49, *p* = 0.023) and miR-155 (HR 1.23; 95%CI 1.06–1.44, *p* = 0.008) were associated with a higher risk of death from the disease. Figure 3A shows Kaplan-Meier estimates for the four miRNAs. Multivariable Cox regression analysis was performed by stratifying patients according to age and using *MGMT* methylation status, *IDH1* mutations, pre-treatment, recurrence, and TCGA prognostic classification as covariates (n = 185). The association with worse OS was confirmed for miR-21 (HR 1.19; 95%CI 1.01–1.41, *p* = 0.04) and miR-210 (HR 1.18; 95%CI 1.02–1.38, *p* = 0.029) (Table S6).

In grade III tumours from the LGG dataset (n = 58), univariable Cox regression analysis demonstrated a significant association with prognosis for 3 of the 13 miRNAs. An association with worse OS was demonstrated with increased expression of miR-155 (HR 1.79; 95%CI 1.26–2.53, *p* = 0.001). Whereas, increased miR-383 (HR 0.56; 95%CI 0.33–0.96, *p* = 0.03) and miR-1296 (HR 0.48; 95%CI 0.26–0.89, *p* = 0.02) was associated with better OS. Figure 3B shows Kaplan-Meier plots for these miRNAs. Multivariable Cox regression analysis was performed including the three miRNAs, age and *IDH1* mutational status. This analysis demonstrated an association between age (HR 1.072; 95%CI 1.015–1.13, p = 0.013) and worse OS, while *IDH1* mutations were associated with better survival (HR 0.014; 0.001–0.216, p = 0.002). These results, however, need to be considered cautiously due to the low number of cases for which complete clinical data were available (n = 51, Table S3b).

None of the selected miRNAs were associated with prognosis in the subgroup of grade II gliomas from the LGG dataset (n = 57). The only clinical parameter associated with survival was the astrocytoma histological subtype (HR 5.81; 95%CI 1.01–33.54, *p* = 0.048) in univariable Cox Regression analysis.

## Discussion

To date, several studies have profiled miRNAs expression in gliomas, but they were mainly performed on GBM cohorts. In this study we compared the miRNA expression profile of 8 WHO grade II gliomas characterized by an overall favourable outcome (median survival 50 months) with 24 WHO grade III and IV tumours characterized by an aggressive clinical behaviour (median survival 16 months). We found 22 miRNAs differentially expressed in the two patients groups; after reassessment by RT-qPCR differential expression between grade II and grade III-IV gliomas was confirmed for 13 out of the 22 miRNAs. The remaining miRNAs were only differentially expressed in gliomas as compared with normal brain tissues. Only one study [11] evaluated the expression of 282 miRNAs in 12 oligodendrogliomas (WHO grade II) and 12 GBMs finding seven miRNAs as differentially expressed between the two tumour types. Interestingly, despite the variance in histotypes between the two works, the 13 miRNAs differentially expressed in our study also include three of the miRNAs (miR-155, miR-21 and miRNA-210) identified by Lages *et al* [11].

To evaluate the prognostic performance of the 13 miRNAs differentially expressed in grade II as compared with grade III+IV gliomas by RT-qPCR, we used the TCGA glioma datasets (GBM and LGG). This analysis demonstrated an association between increased expression and GBM prognosis for four miRNAs: miR-155, miR-21, miR-210, and miR-22. Interestingly, these miRNAs belong to the subgroup of 9 miRNAs differentiating grade II gliomas from grade III+IV tumours in the Venn diagram generated by the analysis of our microarray data, further supporting their biological relevance. The examination of grade III tumours from the LGG dataset identified three miRNAs (miR-155, miR-383, and miR-1296) able to predict patients' survival. Increased miR-155 expression was associated with worse OS in both grade III and grade IV gliomas. Whereas, increased miR-383 expression was associated with a lower risk of death only in grade III tumours. miR-1296 higher expression levels were also found associated with better prognosis in grade III gliomas but no information were available about its prognostic correlation with GBMs, since it was not included on the Agilent platform used to analyze the GBM dataset.

As shown in Table S7, the 6 miRNAs associated with prognosis in this study are involved in all steps of cancer development and progression. We focused our attention on miR-21 and miR-210 because they were the only miRNAs associated with survival in both univariable and multivariable analyses in the GBM dataset and play a pivotal role in hypoxia-related pathways. Indeed, the main histopathological characteristics distinguishing GBMs from lower grade astrocytomas are the presence of foci of necrosis surrounded by hypercellular regions (pseudopalisades), and microvascular hyperplasia that are all hallmarks of hypoxia [12]. This biological phenomenon has been related to aggressiveness and infiltrative behaviour in a wide variety of solid tumours [13]. In GBM, gene expression profiles on microdissected cellular zones surrounding necrotic foci have revealed up regulation of HIF-1α, one of hypoxia-inducible factors mediating cancer cell adaptation to a hypoxic tumour environment [12]. Overexpression of miR-21 in DU145 cells increases the expression of HIF-1α and VEGF and promotes tumor angiogenesis [14]. Interestingly, Hermansen *et al* [15] demonstrated by ISH analysis miR-21 expression in glioma and endothelial cells within the tumor, whereas no expression was detected in non-neoplastic blood vessels. These results are consistent with the hypothesis that miR-21 increased expression is related to tumor angiogenesis in gliomas. On the other side, miR-210 is robustly induced by hypoxia in many cell lines through HIF-1α [16]. This activation determines downregulation of a number of genes which promote cancer cell survival and adaptation to adverse microenvironment, leading to the development of a more resistant and aggressive cell subpopulations. The downregulation of COX10 produces repression of electron transport chain to decrease oxygen-demand, and enhances anaerobic production of ATP via glicolysis enabling survival to hypoxic insults [17]. Upregulation of miR-210 also causes repression of c-Myc inhibitor, MNT, promoting cell cycle and growth [18]. It also contributes to maintenance of high HIF-1α levels during hypoxia, by suppressing of ISCU, SDHS, NUDFA4 proteins, in a feed-forward loop which sustains HIF-1α and its triggered processes [19]. In addition hypoxia-induced miR-210 up-regulation seems to be able to directly enhance tumour angiogenesis by targeting EphrinA3 and ROD1 [20], [21].

## Conclusions

In this study we show that the comparison of grade II and grade III+IV gliomas is able to identify miRNAs distinguishing tumours with a more favourable clinical behaviour from those characterized by a clinical aggressiveness. The validation of our miRNAs panel in the glioblastoma TCGA dataset suggests a direct involvement of miR-21 and miR-210 in glioma development and evolution. Since both miRNAs are involved in mechanisms inducing cancer cell adaptation to hypoxic conditions occurring in rapidly growing and invasive tumours, the development of reliable methods to specifically silence them in cancer cell and/or the identification of agents capable of blocking their downstream effectors may provide new therapeutic strategies for treating this deadly disease.

## Supporting Information

Table S1 **List of miRNAs expression analysis assays.**
(DOCX)

Table S2 **Primer sequences and amplification conditions for IDH1 and TP53 sequencing analyses.**
(DOCX)

Table S3 **Clinicopathological characteristics of TCGA Lower Grade Gliomas (LGG) datasets.** a) Glioblastoma (GBM); b) Lower Grade Glioma (LGG) Grade III; c) Lower Grade Glioma Grade II.
(XLS)

Table S4 **Clinicopathological characteristics of Affymetrix Gene Chip analysis Patients cohort.**
(XLSX)

Table S5 **miRNAs differentially expressed in Affymetrix GeneChip array analysis among Normal Brain Tissue (NBT), Grade II and Grade III+IV gliomas.**
(DOC)

Table S6 **Multivariable Cox regression analysis performed by stratifying patients according to age and using** ***MGMT*** **methylation status,** ***IDH1*** **mutations, pre-treatment, recurrence, TCGA prognostic classification, miR-21, miR-210, miR-22, miR-155, as covariates (n = 185).**
(DOC)

Table S7 **Function of the six miRNAs associated with prognosis by analysis of TCGA datasets.**
(DOC)
