# Supplementary material for: A MiRNA Signature for Defining Aggressive Phenotype and Prognosis in Gliomas
Source: PLoS One. 2014 Oct 3;9(10):e108950. doi: 10.1371/journal.pone.0108950 (PMC4184816; doi:10.1371/journal.pone.0108950)
Supplement: Table S1 — List of miRNAs expression analysis assays. (DOCX) [file pone.0108950.s001.docx]

**Table S1**. List of miRNAs expression analysis assays.

| **ID** | **miR sequence** | **LifeTech Assay P/N** |
| --- | --- | --- |
| *hsa-miR-767-5p_st* | UGCACCAUGGUUGUCUGAGCAUG | 001993 |
| *hsa-miR-105_st* | UCAAAUGCUCAGACUCCUGUGGU | 002167 |
| *hsa-miR-519d_st* | CAAAGUGCCUCCCUUUAGAGUG | 002403 |
| *hsa-miR-155_st* | UUAAUGCUAAUCGUGAUAGGGGU | 002623 |
| *hsa-miR-548j_st* | AAAAGUAAUUGCGGUCUUUGGU | 002783 |
| *hsa-miR-451_st* | AAACCGUUACCAUUACUGAGUU | 001141 |
| *hsa-miR-184_st* | UGGACGGAGAACUGAUAAGGGU | 000485 |
| *hsa-miR-1296_st* | UUAGGGCCCUGGCUCCAUCUCC | 002908 |
| *hsa-miR-21-star_st* | CAACACCAGUCGAUGGGCUGU | 002438 |
| *hsa-miR-629-star_st* | GUUCUCCCAACGUAAGCCCAGC | 001562 |
| *hsa-miR-16_st* | UAGCAGCACGUAAAUAUUGGCG | 000391 |
| *hsa-miR-21_st* | UAGCUUAUCAGACUGAUGUUGA | 000397 |
| *hsa-miR-493_st* | UGAAGGUCUACUGUGUGCCAGG | 002364 |
| *hsa-miR-219-2-3p_st* | AGAAUUGUGGCUGGACAUCUGU | 002390 |
| *hsa-let-7d-star_st* | CUAUACGACCUGCUGCCUUUCU | 001178 |
| *hsa-miR-342-3p_st* | AGAAUUGUGGCUGGACAUCUGU | 002260 |
| *hsa-miR-22_st* | AAGCUGCCAGUUGAAGAACUGU | 000398 |
| *hsa-miR-383_st* | AGAUCAGAAGGUGAUUGUGGCU | 000573 |
| *hsa-miR-210_st* | CUGUGCGUGUGACAGCGGCUGA | 000512 |
| *hsa-miR-223_st* | UGUCAGUUUGUCAAAUACCCCA | 002295 |
| *hsa-miR-326_st* | CCUCUGGGCCCUUCCUCCAG | 000542 |
| *hsa-miR-139-3p_st* | GGAGACGCGGCCCUGUUGGAGU | 002313 |
