# Supplementary material for: A MiRNA Signature for Defining Aggressive Phenotype and Prognosis in Gliomas
Source: PLoS One. 2014 Oct 3;9(10):e108950. doi: 10.1371/journal.pone.0108950 (PMC4184816; doi:10.1371/journal.pone.0108950)
Supplement: Table S2 — Primer sequences and amplification conditions for IDH1 and TP53 sequencing analyses. (DOCX) [file pone.0108950.s002.docx]

**Table S2**. Primer sequences and amplification conditions for IDH1 and TP53 sequencing analyses.

| **Primer ID** | **Primer sequence (5’->3’)** | **PCR annealing temperature (°C)** |
| --- | --- | --- |
| *IDH1 codon 132 for*  *IDH1 codon 132 rev* | CGGTCTTCAGAGAAGCCATT  GTTGGCAATAATGTGATTTTGCATG | 58 |
| *IDH2 codon 172 for*  *IDH2 codon 172 rev* | ATGGCGGCTGCAGTGGG  GAGGGGATCCACTGACCTG | 60 |
| *TP53 exon 5 for*  *TP53 exon 5 rev* | CTTGTGCCCTGACTTTCAACTCTGTCTG  TGGAGAGACGACAGGGCTGGTTGCCCA | 64 |
| *TP53 exon 6 for*  *TP53 exon 6 rev* | CCAGGCCTCTGATTCCTCACTGATTCCTC  GAGGGGTTAAGGGGTGGTTGTCAGTGGC | 64 |
| *TP53 exon 7 for*  *TP53 exon 7 rev* | GCCTCATCTTGGGCCTGTGTTATCTCC  GAGCCACTTGCCACCCTGCACACTGGCC | 64 |
| *TP53 exon 8 for*  *TP53 exon 8 rev* | GTAGGACCTGATTTCCTTACTGCCTCTTGC  GCGGTGGAGGAGACCAAGGGTGCAGTTAT | 66 |
