# Supplementary material for: A MiRNA Signature for Defining Aggressive Phenotype and Prognosis in Gliomas
Source: PLoS One. 2014 Oct 3;9(10):e108950. doi: 10.1371/journal.pone.0108950 (PMC4184816; doi:10.1371/journal.pone.0108950)
Supplement: Table S5 — miRNAs differentially expressed in Affymetrix GeneChip array analysis among Normal Brain Tissue (NBT), Grade II and Grade III+IV gliomas. (DOC) [file pone.0108950.s005.doc]

**Table S5.** miRNAs differentially expressed in Affymetrix GeneChip array analysis among Normal Brain Tissue (NBT), Grade II and Grade III+IV gliomas.

| **Grade II vs NBT (n=80)** | | **Grade III+IV vs NBT (n=71)** | | **Grade II vs Grade III+IV (n=22)** | |
| --- | --- | --- | --- | --- | --- |
| **ID** | ***p*-value** | **ID** | ***p*-value** | **ID** | ***p*-value** |
| hsa-miR-106a_st | 8,34E-05 | hsa-miR-10b_st | 2,89E-06 | hsa-miR-155_st | 1,40E-06 |
| hsa-miR-1226_st | 3,02E-05 | hsa-miR-122_st | 3,28E-07 | hsa-miR-16_st | 5,79E-05 |
| hsa-miR-1272_st | 6,77E-05 | hsa-miR-124_st | 0,000529 | hsa-miR-210_st | 0,000259 |
| hsa-miR-127-3p_st | 0,000255 | hsa-miR-124-star_st | 0,00045 | hsa-miR-219-2-3p_st | 0,00011 |
| hsa-miR-130a_st | 0,000194 | hsa-miR-1250_st | 1,41E-06 | hsa-miR-22_st | 0,000163 |
| hsa-miR-133a_st | 0,000617 | hsa-miR-1258_st | 0,000499 | hsa-miR-223_st | 0,000261 |
| hsa-miR-133b_st | 0,000367 | hsa-miR-1293_st | 0,000341 | hsa-miR-493_st | 6,18E-05 |
| hsa-miR-134_st | 6,06E-05 | hsa-miR-129-3p_st | 0,000325 | hsa-miR-519d_st | 9,19E-07 |
| hsa-miR-135a-star_st | 2,50E-05 | hsa-miR-129-5p_st | 0,000284 | hsa-miR-629-star_st | 4,87E-05 |
| hsa-miR-154-star_st | 4,14E-05 | hsa-miR-138-1-star_st | 6,30E-05 | hsa-let-7d-star_st | 0,000154 |
| hsa-miR-17_st | 0,000108 | hsa-miR-139-5p_st | 0,000218 | hsa-miR-105_st | 8,88E-07 |
| hsa-miR-181a-2-star_st | 2,31E-05 | hsa-miR-206_st | 2,22E-05 | hsa-miR-1296_st | 1,82E-05 |
| hsa-miR-187_st | 0,000388 | hsa-miR-23a_st | 3,88E-06 | hsa-miR-139-3p_st | 0,000297 |
| hsa-miR-195-star_st | 0,000126 | hsa-miR-29b-2-star_st | 0,000141 | hsa-miR-184_st | 2,72E-06 |
| hsa-miR-19a_st | 0,000311 | hsa-miR-338-5p_st | 0,000116 | hsa-miR-21_st | 5,99E-05 |
| hsa-miR-19b_st | 0,000367 | hsa-miR-375_st | 3,21E-06 | hsa-miR-21-star_st | 2,15E-05 |
| hsa-miR-218_st | 0,000667 | hsa-miR-425-star_st | 0,000204 | hsa-miR-326_st | 0,000269 |
| hsa-miR-221_st | 0,000578 | hsa-miR-431-star_st | 3,51E-06 | hsa-miR-342-3p_st | 0,000163 |
| hsa-miR-222_st | 9,93E-05 | hsa-miR-453_st | 0,00044 | hsa-miR-383_st | 0,000233 |
| hsa-miR-299-5p_st | 4,23E-05 | hsa-miR-490-5p_st | 1,74E-07 | hsa-miR-451_st | 2,36E-06 |
| hsa-miR-29c-star_st | 0,000423 | hsa-miR-504_st | 3,22E-07 | hsa-miR-548j_st | 1,69E-06 |
| hsa-miR-324-5p_st | 1,84E-05 | hsa-miR-512-3p_st | 0,000311 | hsa-miR-767-5p_st | 4,35E-07 |
| hsa-miR-331-3p_st | 0,000119 | hsa-miR-516b_st | 0,000499 |  |  |
| hsa-miR-378_st | 0,000722 | hsa-miR-518c-star_st | 4,33E-05 |  |  |
| hsa-miR-379_st | 0,000194 | hsa-miR-584_st | 4,19E-06 |  |  |
| hsa-miR-382_st | 0,00037 | hsa-miR-627_st | 8,64E-05 |  |  |
| hsa-miR-409-3p_st | 0,000251 | hsa-miR-744-star_st | 0,000338 |  |  |
| hsa-miR-409-5p_st | 0,000471 | hsa-miR-885-5p_st | 7,43E-06 |  |  |
| hsa-miR-411_st | 4,20E-05 | hsa-miR-105_st | 0,000188 |  |  |
| hsa-miR-411-star_st | 0,000161 | hsa-miR-1180_st | 1,75E-05 |  |  |
| hsa-miR-425_st | 0,000185 | hsa-miR-1296_st | 0,000599 |  |  |
| hsa-miR-431_st | 2,09E-06 | hsa-miR-129-star_st | 3,75E-06 |  |  |
| hsa-miR-432_st | 8,67E-05 | hsa-miR-1301_st | 1,56E-06 |  |  |
| hsa-miR-487a_st | 0,000533 | hsa-miR-132_st | 6,37E-07 |  |  |
| hsa-miR-495_st | 0,000573 | hsa-miR-138-2-star_st | 8,91E-10 |  |  |
| hsa-miR-497_st | 0,00011 | hsa-miR-139-3p_st | 2,40E-08 |  |  |
| hsa-miR-500_st | 0,000561 | hsa-miR-149_st | 0,000118 |  |  |
| hsa-miR-542-5p_st | 4,74E-07 | hsa-miR-184_st | 3,35E-07 |  |  |
| hsa-miR-543_st | 9,78E-05 | hsa-miR-21_st | 0,000509 |  |  |
| hsa-miR-628-3p_st | 0,000173 | hsa-miR-212_st | 0,000222 |  |  |
| hsa-miR-629_st | 0,000574 | hsa-miR-21-star_st | 1,79E-05 |  |  |
| hsa-miR-654-5p_st | 5,51E-05 | hsa-miR-25_st | 0,000289 |  |  |
| hsa-miR-768-3p_st | 2,03E-06 | hsa-miR-323-3p_st | 3,69E-05 |  |  |
| hsa-miR-768-5p_st | 0,000148 | hsa-miR-323-5p_st | 1,29E-05 |  |  |
| hsa-miR-769-5p_st | 0,000411 | hsa-miR-326_st | 7,03E-08 |  |  |
| hsa-miR-99a_st | 7,78E-07 | hsa-miR-328_st | 2,59E-06 |  |  |
| hsa-miR-99a-star_st | 4,74E-05 | hsa-miR-330-3p_st | 3,40E-05 |  |  |
| hsa-let-7d-star_st | 0,000131 | hsa-miR-330-5p_st | 7,25E-08 |  |  |
| hsa-miR-1180_st | 0,000636 | hsa-miR-342-3p_st | 0,000286 |  |  |
| hsa-miR-129-star_st | 4,21E-05 | hsa-miR-342-5p_st | 0,000513 |  |  |
| hsa-miR-1301_st | 0,00018 | hsa-miR-346_st | 2,20E-05 |  |  |
| hsa-miR-132_st | 0,000485 | hsa-miR-370_st | 0,000473 |  |  |
| hsa-miR-138-2-star_st | 0,000191 | hsa-miR-383_st | 5,76E-06 |  |  |
| hsa-miR-139-3p_st | 5,93E-05 | hsa-miR-432-star_st | 6,53E-06 |  |  |
| hsa-miR-149_st | 6,31E-05 | hsa-miR-433_st | 1,95E-06 |  |  |
| hsa-miR-212_st | 0,000455 | hsa-miR-451_st | 7,71E-05 |  |  |
| hsa-miR-25_st | 0,000132 | hsa-miR-485-3p_st | 5,48E-06 |  |  |
| hsa-miR-323-3p_st | 0,000229 | hsa-miR-485-5p_st | 3,53E-06 |  |  |
| hsa-miR-323-5p_st | 2,03E-07 | hsa-miR-491-5p_st | 0,000162 |  |  |
| hsa-miR-328_st | 5,42E-05 | hsa-miR-519a-star_st | 1,02E-07 |  |  |
| hsa-miR-330-3p_st | 0,000183 | hsa-miR-519c-5p_st | 4,02E-07 |  |  |
| hsa-miR-330-5p_st | 0,000181 | hsa-miR-548j_st | 2,30E-09 |  |  |
| hsa-miR-342-5p_st | 2,13E-05 | hsa-miR-668_st | 6,79E-06 |  |  |
| hsa-miR-346_st | 1,52E-05 | hsa-miR-744_st | 5,07E-05 |  |  |
| hsa-miR-370_st | 0,000128 | hsa-miR-766_st | 2,81E-06 |  |  |
| hsa-miR-432-star_st | 6,03E-06 | hsa-miR-767-5p_st | 0,000388 |  |  |
| hsa-miR-433_st | 0,000252 | hsa-miR-769-3p_st | 1,70E-05 |  |  |
| hsa-miR-485-3p_st | 8,13E-05 | hsa-miR-770-5p_st | 2,39E-09 |  |  |
| hsa-miR-485-5p_st | 0,000438 | hsa-miR-873_st | 2,57E-10 |  |  |
| hsa-miR-491-5p_st | 0,00029 | hsa-miR-874_st | 1,99E-08 |  |  |
| hsa-miR-519a-star_st | 3,70E-05 | hsa-miR-935_st | 8,15E-05 |  |  |
| hsa-miR-519c-5p_st | 2,85E-05 |  |  |  |  |
| hsa-miR-668_st | 2,14E-05 |  |  |  |  |
| hsa-miR-744_st | 1,10E-05 |  |  |  |  |
| hsa-miR-766_st | 7,99E-07 |  |  |  |  |
| hsa-miR-769-3p_st | 8,21E-06 |  |  |  |  |
| hsa-miR-770-5p_st | 0,000119 |  |  |  |  |
| hsa-miR-873_st | 3,02E-07 |  |  |  |  |
| hsa-miR-874_st | 5,38E-05 |  |  |  |  |
| hsa-miR-935_st | 0,000546 |  |  |  |  |
