# Supplementary material for: A MiRNA Signature for Defining Aggressive Phenotype and Prognosis in Gliomas
Source: PLoS One. 2014 Oct 3;9(10):e108950. doi: 10.1371/journal.pone.0108950 (PMC4184816; doi:10.1371/journal.pone.0108950)
Supplement: Table S6 — Multivariable Cox regression analysis performed by stratifying patients according to age and using MGMT methylation status, IDH1 mutations, pre-treatment, recurrence, TCGA prognostic classification, miR-21, miR-210, miR-22, miR-155, as covariates (n = 185). (DOC) [file pone.0108950.s006.doc]

**Supplemental Table 6.** Multivariable Cox regression analysis performed by stratifying patients according to age and using *MGMT* methylation status, *IDH1* mutations, pre-treatment, recurrence, TCGA prognostic classification, miR-21, miR-210, miR-22, miR-155, as covariates (n=185).

| **Variable** | **HR** | **95% CI** | **P value** |
| --- | --- | --- | --- |
| **MGMT unmethylated** | 1.38 | 0.97-1.98 | 0.07 |
| **IDH1 mutation** | 1.02 | 0.55-1.86 | 0.96 |
| **No Treatment** | 20.50 | 2.20-190.5 | **0.008** |
| **No Recurrence** | 0.12 | 0.01-1.09 | 0.06 |
| **TCGA classification** |  |  |  |
| Astro | 0.75 | 0.439-1.27 | 0.28 |
| Neural | 1.12 | 0.65-1.92 | 0.69 |
| Neuromesenchimal | 0.79 | 0.49-1.26 | 0.33 |
| Oligoneural | 0.75 | 0.46-1.24 | 0.26 |
| **miR-21** | 1.23 | 1.03-.1.48 | **0.02** |
| **miR-210** | 1.21 | 1.02-1.43 | **0.03** |
| **miR-22** | 0.90 | 0.67-1.21 | 0.49 |
| **miR-155** | 1.13 | 0.91-1.39 | 0.26 |
| **miR-219** | 0.97 | 0.87-1.08 | 0.54 |
| **miR-223** | 1.01 | 0.80-1.27 | 0.56 |
