# Supplementary material for: A MiRNA Signature for Defining Aggressive Phenotype and Prognosis in Gliomas
Source: PLoS One. 2014 Oct 3;9(10):e108950. doi: 10.1371/journal.pone.0108950 (PMC4184816; doi:10.1371/journal.pone.0108950)
Supplement: Table S7 — Function of the six miRNAs associated with prognosis by analysis of TCGA datasets. (DOC) [file pone.0108950.s007.doc]

**Table S7.** Function of the six miRNAs associated with prognosis by analysis of TCGA datasets.

| **miRNA** | **Expression level** | **Function** | **Targets** | **Prognostic value** | **References** |
| --- | --- | --- | --- | --- | --- |
| **miR-210** | ***Up*** in NSCLC, breast cancer, head and neck cancee (HNSCC), pancreatic cancer, malignant melanoma, soft-tissue sarcoma, renal cell carcinoma and glioblastoma. ***Down*** in gastric cancer. ***Deleted*** in epithelial ovarian cancer. | oncogene | Bcl-2, P4HB, PTPN1, MDGA1, MID1IP1, APC, CLASP2, PTAR1, TNPO1, CBX1, CHD9, SMCHD1, XIST, NIPBL, RAD52, CPEB2, EFNA3, ELK3, NPTX1, MIB1, HOXA1/3/9, HECTD1, ISCU, ATP11C, DDAH1, GPD1L, ACVR1b, BDNF, MNT, E2F3, SEH1L, FGFRL1, CASPSAP2, ROD1 | Gliomas: lower expression level in ODG vs GBM (ANOVA, *p*< 0.05) [10] ; MiR210 low expression levels correlate with long OS (p=0.0077) and long PFS survival (p=0.0212) [8]. Breast cancer: upregulation associates with poor overall survival (HR=3.39, *p*< 0.05) [11].  HNSCC: the level of hsa-miR-210 results significantly correlated with the 99-gene hypoxia metagene (Spearman rank test rho=0.67, *p*< .001) and high levels of hsa-miR-210 are associated with locoregional disease recurrence (Univariate Kaplan Meier analysis*, p*=0.003) and short OS (Univariate Kaplan Meier analysi,s *p*=0.008) [12]. | **10.** MicroRNA and target protein patterns reveal physiopathological features of Glioma subtypes. Lages E, Guttin A, El Atifi *et al*. PLoS ONE (**2011**) 6:5. **11.** High expression of miR-210 predicts poor survival in patients with Breast Cancer:A meta-analysis. Hong L, Yang J *et al*. Gene 507 (**2012**) 135–138. **12.** hsa-mir-210 is a marker of tumor hypoxia and a prognostic factor in Head and Neck Cancer. Gee H.E, Camps C, Buffa F.M *et al*. Cancer (**2010**). |
| **miR-21** | ***Up*** in lung and breast cancers, pancreas, stomach, prostate tumours, CLL, acute myeloid leukemia (AML), osteosaroma, colon cancer, myeloma, glioblastoma, in hepatocellular carcinoma (HCC), head and neck squamous cell carcinoma (HNSCC). | oncogene | PTEN, TPM1, PDCD4, SPRY1/2, TIMP3, RECK, RECK, TGFBR, DAXX, PDCD4, p63, JMY, TOPORS, HNRNPK, and TP53BP2 | Gliomas: miR-21 levels correlated significantly with grade (*p*=0.027) and inversely correlated with overall survival in all grade (*p*=0.005) [13]. Plasma levels of miR-21 are significantly higher in GBM samples than in control samples (Mann–Whitney U test*, p* < 0.001) with a 90% sensitivity and a 100% specificity (ROC curve analysis, AUC=0.9300, 95% CI: 0.7940-1.066) [14]. NSCLC: overexpression of mature miR-21 is found to be an independent prognostic factor for OS (Multivariate analysis: hazard ratio 2.533, CI 1.066–6.020, *p=*0.035) [15]. High levels of circulating miR-21 were predictive of the ER-negative status in breast cancer and docetaxel resistance in prostate cancer [9]. Colon adenocarcinoma**:** High miR-21 expression was associated with poor survival and poor therapeutic outcome [16]. HCC: high level of miR-21 are correlated with frequency of coexisting cirrhosis (*p* < 0.0001), T stage progression (*p*=0.02) and poor OS (Univariate Kaplan Meier analysis, *p*=0.036) [17] | **13.** MiR-21 expression in the tumor cell compartment holds unfavorable prognostic value in Gliomas. Hermansen S *et al*. J Neurooncol (**2013**) 111:71–81. **14**. Plasma specific miRNAs as predictive biomarkers for diagnosis and prognosis of Glioma. Wang Q, Li P, Li A *et al*. Journal of Experimental & Clinical Cancer Research (**2012**), 31:97. **15.** Prognostic value of mature microRNA-21 and microRNA-205 overexpression in Non–Small Cell Lung Cancer by Quantitative Real-Time RT-PCR. Markou A, Tsaroucha E.G *et al*. Clinical Chemistry (**2008**) 54:10 1696–1704. **16.** MicroRNA-21 gene and cancer. Huang Y *et al*. Med Oncol (**2013)** 30:376. **17.** Expression of microRNAs, miR-21, miR-31, miR-122, miR-145, miR-146a, miR-200c, miR-221, miR-222, and miR-223 in patients with Hepatocellular Carcinoma or Intrahepatic cholangiocarcinoma and its prognostic significance. Karakatsanis A, Papaconstantinou I, Gazouli M, *et al*. Molecular Carcinogenesis (**2013**) 52:297–303 |
| **miR-22** | ***Down*** in breast cancer, lung, gastric and colorectal cancer, multiple myeloma, cholangiocarcinoma, hepatocellular carcinoma (HCC). ***Up*** in brain metastases from colon cancer. | tumor-suppressor | estrogen receptor α (ERα), histone deacetylase 4 (HDAC4), CDK6, SIRT1, Sp1, p21, hypoxia inducible factor 1α (HIF-1α), MYCBP and MAX, PTEN | Colorectal cancer: Downregulation correlates with low OS (Univariate Kaplan Meier analysis, *p* =0.003) and liver metastases (HR= 7.624) [1]. HCC: Downregulation correlates with reduced DFS (Univariate Kaplan Meier analysis*, p*=0.025) [2]. | **1.** Clinical significance of miR-22 expression in patients with Colorectal Cancer. Zhang G, Xia S, Tian H, *et al*. Med Oncol (**2012**) 29:3108–3112. **2.** Emerging Roles of microRNA-22 in human disease and normal physiology. Xiong J. Current Molecular Medicine (**2012**) 12:247-258. |
| **miR-155** | ***Up*** in leukemias/ lymphomas, breast and colon cancer, thyroid, lung, pancreatic, liver and gastric tumors and gliomas. | oncogene | MYD88,FADD, RHOA, SMAD2, METS1, ETS1, SPI1, CEBPB, MECP2, FOXO3, MECP2, SOCS1, BACH1, IKBKE, FOXO3A, hMSH2, hMSH6, hMLH1, SMAD5, WEE1, SHIP1, TNF, IL-8, FGF, SEL1L | Gliomas: miR-155 low expression levels correlate with long OS (Univariate Kaplan Meier analysis, *p*=0.0191) and long PFS survival (Univariate Kaplan Meier analysis*, p*=0.0055) [8] . AML: upregulation of miR-155 is strongly but independently predictive of FLT3 gene mutations (*p=*0.01) [9] . Breast cancer: High levels of miR-155 are associated to a positive progesterone receptor status [9]. | **8.** Interactions of miR-323/miR-326/miR-329 and miR-130a/miR-155/miR-210 as prognostic indicators for clinical outcome of Glioblastoma patients. Qiu S, Lin S, Hu D, *et al*. Journal of Translational Medicine (**2013)**, 11,10. **9.** miRNA profiling of cancer. Gianpiero Di Leva and Carlo M Croce. Current Opinion in Genetics & Development (**2013)**, 23:1–9. |
| **miR-1296** | ***Down*** in prostate cancer | tumor-suppressor | MCM2 |  | **18.** Regulation of minichromosome maintenance gene family by microRNA-1296 and genistein in Prostate Cancer. Shahana Majid, Altaf A. Dar, Sharanjot Saini, *et al.* Cancer Res (**2010**);70:2809-2818. |
| **miR-383** | ***Down*** in gliomas, medulloblastoma (MB), ependymomas, human  testicular embryonal carcinoma, hepatocellular carcinoma, breast and ovarian cancers, melanoma, acute myeloid leukemia.  ***Up*** in Clear Cell Renal Cell Carcinoma (ccRCC) | tumor-suppressor  oncogene | IGFR1, PRDX3, DIO1, RBMS1, IRF1 |  | **19.** Downregulation of miR-383 promotes glioma cell invasion by targeting insulin-like growth factor 1 receptor. Zhanwen He, Danyang Cen, Xiangyang Luo *et al*. Med Oncol (**2013**) 30:557. **20.** Identification of microRNAs as potential prognostic markers in Ependymoma. Fabricio F. Costa, Jared M. Bischof, Elio F. Vanin, *et al* PLoS ONE (**2011**), 6, 10.  **21.** MiR-224 targets the 3'UTR of Type 1 5'-Iodothyronine Deiodinase possibly contributing to tissue hypothyroidism in Renal Cancer. J. Boguslawska, A. Wojcicka, A. Piekielko-Witkowska, *et al.* PLoS ONE (**2011**), 6, 9. |
